# Modified constraint-induced movement therapy or bimanual occupational therapy following injection of Botulinum toxin-A to improve bimanual performance in young children with hemiplegic cerebral palsy: a randomised controlled trial methods paper

**DOI:** 10.1186/1471-2377-10-58

**Published:** 2010-07-05

**Authors:** Brian J Hoare, Christine Imms, Hyam Barry Rawicki, Leeanne Carey

**Affiliations:** 1 School of Occupational Therapy, La Trobe University, Bundoora, 3086, Australia; 2 Victorian Paediatric Rehabilitation Service, Monash Medical Centre, 246 Clayton Road, Clayton, 3168, Australia; 3 Murdoch Children's Research Institute, Melbourne, Australia; 4 Royal Children's Hospital, Melbourne, Australia; 5 National Stroke Research Institute, Florey Neuroscience Institutes, Melbourne, Australia

## Abstract

**Background:**

Use of Botulinum toxin-A (BoNT-A) for treatment of upper limb spasticity in children with cerebral palsy has become routine clinical practice in many paediatric treatment centres worldwide. There is now high-level evidence that upper limb BoNT-A injection, in combination with occupational therapy, improves outcomes in children with cerebral palsy at both the body function/structure and activity level domains of the International Classification of Functioning, Disability and Health. Investigation is now required to establish what amount and specific type of occupational therapy will further enhance functional outcomes and prolong the beneficial effects of BoNT-A.

**Methods/Design:**

A randomised, controlled, evaluator blinded, prospective parallel-group trial. Eligible participants were children aged 18 months to 6 years, diagnosed with spastic hemiplegic cerebral palsy and who were able to demonstrate selective motor control of the affected upper limb. Both groups received upper limb injections of BoNT-A. Children were randomised to either the modified constraint-induced movement therapy group (experimental) or bimanual occupational therapy group (control). Outcome assessments were undertaken at pre-injection and 1, 3 and 6 months following injection of BoNT-A. The primary outcome measure was the Assisting Hand Assessment. Secondary outcomes included: the Quality of Upper Extremity Skills Test; Pediatric Evaluation of Disability Inventory; Canadian Occupational Performance Measure; Goal Attainment Scaling; Pediatric Motor Activity Log; modified Ashworth Scale and; the modified Tardieu Scale.

**Discussion:**

The aim of this paper is to describe the methodology of a randomised controlled trial comparing the effects of modified constraint-induced movement therapy (a uni-manual therapy) versus bimanual occupational therapy (a bimanual therapy) on improving bimanual upper limb performance of children with hemiplegic cerebral palsy following upper limb injection of BoNT-A. The paper outlines the background to the study, the study hypotheses, outcome measures and trial methodology. It also provides a comprehensive description of the interventions provided.

**Trial Registration:**

ACTRN12605000002684

## Background

Cerebral palsy is "a group of permanent disorders of the development of movement and posture causing activity limitation(s) that are attributed to non-progressive disturbances that occurred in the developing fetal or infant brain" [[1], p. 9]. Secondary to the brain disturbance, children with cerebral palsy often experience neurological symptoms including dystonia, ataxia, athetosis and particularly spasticity [2]. Spasticity occurs as a result of a loss of upper motor neuron inhibition on the lower motor neurons which results in increased or impaired motor unit firing and altered muscle tone [2]. Muscle spasticity is characterised by a velocity-dependent increase in tonic stretch reflexes (muscle tone) with exaggerated tendon jerks (phasic stretch reflex) resulting from hyperexcitability of the stretch reflex [3]. Adding to these neurological symptoms, skeletal muscle morphology in children with cerebral palsy is also altered due to abnormally long muscle sarcomere lengths and muscle tissue containing a hypertrophic extracellular matrix of poor quality [2,4]. This results in muscle stiffness affecting posture and movement and can be described as hypertonia or increased muscle tone [3].

Hemiplegic cerebral palsy, characterised by a clinical pattern of unilateral motor impairment, accounts for 35.1% of all cerebral palsy types in Victoria, Australia [5], 15.3% in Ontario, Canada, 40% in Sweden [6] and 31.2% in North England, United Kingdom [7]. Along with muscle spasticity and hypertonia, children with hemiplegic cerebral palsy experience a loss of upper motor neurone excitation that is typically associated with poor selective motor control and weakness, and in some instances, sensory deficits. These additional impairments significantly impact on a child's ability to perform daily tasks [8-11].

The spastic motor type of cerebral palsy is the most common, comprising about 80% of all reported cases [12]. Although the mechanism is unknown, spastic muscle often shortens to create muscle contractures, which often leads to fixed deformity and further functional complications [13]. Therefore, management of the upper limb in children with cerebral palsy usually involves a variety of interventions targeting the musculoskeletal system. These may include splinting and casting, passive stretching, the facilitation of posture and movement (e.g. occupational therapy and physiotherapy) or systemic spasticity-reducing medication and surgery [14]. Botulinum toxin-A (BoNT-A) is now commonly used as an adjunct to these interventions.

### Botulinum toxin-A in the treatment of the upper limb in children with cerebral palsy

BoNT-A is a powerful neuromuscular paralysing agent that is produced by the anaerobic bacterium *clostridium botulinum *[15]. BoNT-A acts at the neuromuscular junction by inhibiting the release of the neurotransmitter *acetylcholine*. Injection of BoNT-A into selected muscles produces dose-dependent chemical denervation resulting in reduced muscle activity. The denervation is temporary as sprouting of new nerve terminals from the treated nerves leads to re-innervation. The function of the original terminal is eventually restored leading to the recovery of the affected muscles [16]. The period of clinically useful relaxation appears to be 12-16 weeks [12]. The aim of BoNT-A in the treatment of the upper limb in children with cerebral palsy is to produce selective reduction in muscle spasticity using the smallest possible dose. The reduction in spasticity is intended to provide an opportunity to optimise the effects of motor training by reducing the negative interference of spasticity. It can also serve to improve tolerance and compliment the effects of splinting and casting potentially delaying the need for soft tissue surgery [9].

Use of BoNT-A for the treatment of upper limb spasticity in children with cerebral palsy has become routine clinical practice in many paediatric treatment centres worldwide. In Australia, injection of BoNT-A (Botox(r) only) is now an approved and government funded treatment for moderate to severe spasticity of the upper limbs children with cerebral palsy, 2 to 17 years of age inclusive [17]. A recent Cochrane review, including ten randomised controlled trials predominantly of high quality, provided high-level evidence that BoNT-A in the upper limb, in combination with occupational therapy, improved outcomes at both the body function/structure and activity level domains of the International Classification of Functioning, Disability and Health (ICF) [18] when compared to occupational therapy alone, BoNT-A alone or no treatment [19]. The review authors concluded that injection of BoNT-A in the upper limb in children with cerebral palsy should always be accompanied by planned post-injection therapeutic intervention. This conclusion is consistent with a recently developed international collaboration documenting upper limb BoNT-A evidence-based guidelines for intervention and follow-up for children with cerebral palsy [20].

Intramuscular injection of BoNT-A results in relaxation of the affected muscle providing a window of opportunity for maximising therapy outcomes. For children with hemiplegia however, injection of BoNT-A alone does not improve the ability of the child to use the affected limb and clinical experience suggests attempts to use the limb immediately following injection often remain effortful, inefficient and result in clumsy movement. Persistent verbal and physical encouragement by therapists and parents may frequently lead to further frustration and negative or defiant behavior. Therapy must, therefore, create the opportunity, motivation, experience and environment in which a child can learn how to use their affected limb to maximize the effectiveness of the BoNT-A.

A recent systematic review of all upper limb interventions in children with cerebral palsy found that occupational therapy in combination with BoNT-A produced the largest treatment effect of all upper limb interventions on activity level outcomes [21]. Despite high-level evidence supporting occupational therapy post-injection, no clinical trial has specifically investigated the optimal type and amount of therapy following BoNT-A. Approaches undertaken in recent trials however, all point to the effectiveness of bursts of therapy focused on specific skill acquisition or goal achievement [22-26]. Further investigation is required to establish what intensity and/or specific type of therapy will enhance functional outcomes and prolong the beneficial effects of BoNT-A [14].

### Movement-based therapies in the upper limb

For occupational therapists, bimanual occupational therapy training (BOT) in children with hemiplegia is a commonly applied intervention. Theories of practice have previously been described in the literature [27-29], although clinical service delivery is eclectic and potentially covers a range of interventions. An essential element of the bimanual approach to upper limb training includes the repetitive practice of motivating, meaningful and purposeful bimanual activities (i.e. occupations). This has been well supported by recent advances in the areas of neuroscience, basic mechanisms of hand function and more specifically, motor control and motor learning theories [30]. Although this approach is commonly used and has strong theoretical foundations, it has not been systematically defined and empirically investigated.

In 2007, Gordon and colleagues comprehensively described and evaluated a bimanual approach to upper limb training in children with hemiplegic cerebral palsy called Hand Arm Bimanual Intensive Training (HABIT) [31]. Despite similarities with BOT, specific differences exist between the therapies. HABIT includes: high intensity of treatment (6 hours per day for 2 weeks); the use of behavioural shaping theory [32]; and relies solely on environmental adaptation for grading of activities, rather than physical assistance or handling of the child (Andrew Gordon, personal communication). Children participating in HABIT demonstrated improved bimanual upper limb function compared with a group receiving customary care [31]. However, the promising evidence provided by this small trial of moderate methodological quality suggests that further investigation of bimanual intervention in children with hemiplegia is both justified and warranted [28].

Constraint-induced movement therapy (CIMT) has been adopted as a method of teaching a child to use his/her affected upper limb through use of a restraint on the non-affected limb and massed practice of movements of the affected limb [33]. It has been proposed as an effective way of improving outcomes following upper limb BoNT-A injection, particularly as it removes the need for persistent prompting [34]. It may, on the other hand, lead to the opposite effect if a child demonstrates adverse behavioral responses to the restraint [33].

*Developmental disregard *is a term used to describe a child with hemiplegia who may disregard, or learn not to use, the affected limb during the development of motor function [35]. Children with even mild impairment in the affected limb learn effective strategies to manage daily tasks (e.g. play) with one hand, discovering that performance is more efficient using only the non-affected hand [10]. Constraint-Induced Movement Therapy (CIMT), aims to reverse the behavioral suppression of movement in the affected upper limb by constraining use of the non-affected limb and providing massed practice of activities with the affected limb [36]. Studies in adults following stroke have provided evidence of adaptation in the brain involving the motor cortical areas controlling movement of the more affected limb following CIMT [37-39]. Since the potential for central nervous system plasticity in young children is increased relative to adults [40-42], it is postulated that this approach might prove to be especially effective in children [43].

CIMT is a multi-faceted intervention and studies describing its use in children with cerebral palsy present wide variation in its application in relation to: method of restraint; length of restraint (per day, number of weeks); type and duration of therapy; intervention environment (that is home, school, or clinic) and intervention provider (therapist, parent, or teacher). Children included in studies have also varied in age, diagnosis, severity of motor and sensory impairment, cognitive abilities and behavior. Despite the emerging popularity of CIMT in children with hemiplegic cerebral palsy, a Cochrane review [44] identified a significant treatment effect in only a single trial which adopted a less intensive modified form of CIMT [8]. The modified CIMT (mCIMT) involved the application of a restraint on the unaffected upper limb and less than three hours per day of therapy provided to the affected limb [44]. While a positive trend was found favoring CIMT [35] and Forced Use [45], no significant treatment effect was demonstrated for these interventions when compared with traditional services.

Studies published to date have provided limited evidence of factors that may impact on the effectiveness of CIMT. Response to CIMT does not appear to be age-dependent and children with more impaired bimanual hand function demonstrate greater improvement than less impaired children [8]. Although not statistically significant, trends have also been reported as favoring children with cortical/subcortical lesions compared to those with periventricular white matter lesions [8]. More recently, Kuhnke et al., [46] reported that adolescents and young adults with ipsilateral corticospinal projections responded differently to CIMT intervention in comparison to those with contralateral projections. Although these findings must be interpreted cautiously due to methodological limitations [47], the study by Kuhnke et al., [46] adds information about children's potential responses to CIMT.

There is currently no published trial directly comparing a BOT protocol with a CIMT protocol. However, in 2008 Gordon and colleagues [48] published data from a quasi-randomised trial of 16 children with hemiplegic cerebral palsy that compared a one-on-one uni-manual approach to treatment (CIMT) versus a one-on-one bimanual approach to treatment (HABIT) [48]. This small trial reported similar changes for both groups on outcomes including the Jebsen-Taylor Test of Hand Function, Assisting Hand Assessment and accelerometry. The finding, measured using the Assisting Hand Assessment, indicated immediate post-treatment improvements in upper limb bimanual performance using either a uni-manual or bimanual approach to training. However, retention of gains was not examined following either intervention.

## Methods/Design

The aim of this current paper is to describe the methodology of a randomised controlled trial comparing the effects of modified constraint-induced movement therapy (a uni-manual therapy) versus conventional bimanual occupational therapy on improving bimanual upper limb performance of children with hemiplegic cerebral palsy following upper limb injection of BoNT-A. Bimanual upper limb performance, assessed using the Assisting Hand Assessment, was selected as the primary measure as the central aim of all upper limb motor-based interventions in children with hemiplegia is to improve a child's actual use of their affected upper limb in a range of daily tasks, including those requiring bimanual performance; rather than what they can do in a clinical setting or how normal their movement appears. This RCT builds on the existing evidence supporting the use of BoNT-A in the upper limb of children with cerebral palsy. It aims to identify the most effective intervention following injection of BoNT-A. A further aim is to explore the individual characteristics of children that impact on response to treatment. Finally, it will also provide additional evidence regarding dose response to treatment, specificity of training effects, and the retention of treatment effects.

### Primary objective

To compare the effects of modified CIMT versus bimanual occupational therapy (BOT) in improving the bimanual performance of children with hemiplegic cerebral palsy following upper limb BoNT-A injection.

#### Hypothesis 1

Modified CIMT is more effective than conventional BOT in improving the bimanual performance of children with hemiplegic cerebral palsy following upper limb BoNT-A injection.

#### Hypothesis 2

Relative to baseline performance on the Assisting Hand Assessment, modified CIMT results in greater retention of gains in bimanual performance compared with BOT.

### Secondary objectives

A. To compare the effect of modified CIMT versus bimanual occupational therapy (BOT) on secondary outcomes relating to: the performance of activities of daily living; the quality of upper limb movement; occupational performance; individual goal achievement; and frequency of use of the affected upper limb of children with hemiplegic cerebral palsy following upper limb BoNT-A injection.

#### Hypothesis 3

Modified CIMT is more effective than conventional BOT in improving performance of activities of daily living, quality of upper limb movement, occupational performance, individual goal achievement and frequency of use of the affected upper limb in children with hemiplegic cerebral palsy following upper limb BoNT-A injection.

B. To evaluate the clinical significance of change identified on the primary outcome, the Assisting Hand Assessment.

#### Hypothesis 4

That changes on the Assisting Hand Assessment associated with intervention are of a clinically significant magnitude. Clinical significance was defined as a change score of more than 5 raw score points (2.6 logits) on the AHA (Lena Krumlinde-Sundholm, personal communication).

C. To evaluate the impact of age and actual treatment dosage on the primary outcome, the Assisting Hand Assessment.

#### Hypothesis 5

Younger children (irrespective of treatment type) will show greater improvement in bimanual performance following uni-manual or bimanual therapy and upper limb BoNT-A injection.

#### Hypothesis 6

Children who undertake treatment of greatest intensity (irrespective of treatment type) will show greater improvement in bimanual performance.

### Study design

Randomised, controlled, evaluator-blinded, prospective parallel-group trial based on Consolidated Standards of Reporting Trials (CONSORT) Statement for Randomised Trials of Non-Pharmacologic Treatment [49].

### Participants

Children were eligible to participate if they met the following criteria:

### Inclusion Criteria

• Diagnosis of congenital spastic hemiplegic cerebral palsy as diagnosed and reported in the medical history by a medical specialist (i.e. neurologist, paediatrician).

• Aged 18 months to 6 years at time of recruitment.

• Active movement of the shoulder, elbow, wrist, digits and thumb of the affected upper limb, such that the:

• child is able to reach forward to an elevated position in front with mid range shoulder flexion.

• child is able to grasp a 2.5 cm cube from a table top and release it in a large container (20 cm × 14 cm).

• Able to attend to tasks and follow simple one stage commands.

• child is able to actively perform reach and grasp/release activities with verbal prompting.

• Moderate levels of muscle tone (i.e. 1-2, modified Ashworth scale) and spasticity (i.e. 1-2, Tardieu scale) and no fixed contracture in target group of muscles to be injected with BoNT-A.

• Parents able to commit to an intensive therapy program and agree to cease all other upper limb therapeutic interventions for the 6-month period of the trial.

• Assessed as appropriate for upper limb BoNT-A at neuromuscular clinic by rehabilitation specialist (BR).

### Exclusion Criteria

Children who otherwise met the inclusion criteria were excluded if they had:

• Congenital quadriplegic or diplegic cerebral palsy.

• Previous BoNT-A injections in the upper limb in the past 12 months.

• Prior upper limb surgery (i.e. tendon transfer/tendon lengthening).

• Existing treatments that are not compatible with those those included in the study treatment package.

There were no criteria relating to exclusion of children with mother tongue other than English, presence of co-morbidity or socio-economic status.

### Recruitment

Potential participants were identified from the Physical Rehabilitation Clinic of a major paediatric metropolitan hospital. In addition, postal and email advertising were sent to local medical practitioners and paediatric therapy networks in metropolitan Melbourne. Potential participants were screened by the chief investigator (BJH) to determine eligibility. Children eligible for inclusion were then assessed by an experienced rehabilitation specialist for suitability for upper limb BoNT-A injection (HBR). Suitable children were then invited to participate in the RCT and informed consent obtained for the participation of the child and the child's parent prior to enrolment in the RCT.

### Procedure

The RCT was approved by the Ethics Committee of both Southern Health and La Trobe University. The trial is registered with the Australian New Zealand Clinical Trials Registry (ACTRN12605000002684) [50]. Data was collected from August 2003 until May 2009.

### Reliability training for assessment and scoring of outcome measures

Prior to commencement of the RCT, two highly experienced paediatric therapists (one occupational therapist, one physiotherapist) were trained in the administration of all outcome measures. A manual was developed for both therapists to ensure consistency in measurement over the recruitment period. The same occupational therapist collected data for the Assisting Hand Assessment, Quality of Upper Extremity Skills Test, Pediatric Evaluation of Disability Inventory, Pediatric Motor Activity Log, Canadian Occupational Performance Measure and Goal Attainment Scaling for the entire recruitment period. Two physiotherapists collected data for the modified Ashworth and modified Tardieu Scales. Scoring of the Assisting Hand Assessment was undertaken by the assessment developers. The Quality of Upper Extremity Skills Test was scored by a highly experienced paediatric occupational therapist who had significant experience in the assessment and treatment of children following upper limb BoNT-A injections.

## Randomisation

### Sequence generation

Children were block randomised into pairs matched by age (± 6 months) using a computer generated set of random numbers.

### Allocation Concealment

A set of random numbers was used to create an allocation sequence which was contained in individual opaque envelopes for use by the chief investigator (BJH). As children were recruited, the next envelope in the sequence was opened and the child assigned to the stated group.

### Implementation

All randomisation, sequence generation, and preparation of group allocation materials were performed by a third party (the Monash Institute of Health Services Research) who had no direct contact with the clinical aspects of the trial. The master list of random numbers was located in locked cabinets at the Institute and only accessible at completion of the RCT for analysis.

### Blinding

Due to the overt nature of the interventions, children, their parents and the treating therapist were not blinded to group assignment, however, children and parents were blind to the RCT hypotheses. Outcome measures were administered by a therapist blind to group assignment and scored by different assessors who were blind to group allocation. The Assisting Hand Assessment and Quality of Upper Extremity Skills Test was videotaped, randomised and scored by assessors blind to group allocation and order of assessment.

### Sample Size

Sample size estimates were based on projected treatment effect on the primary outcome measure, the Assisting Hand Assessment. In 2005, the authors suggested that a change of more than 5 raw score points may represent a clinically meaningful difference on the Assisting Hand Assessment (Lena Krumlinde-Sundholm, personal communication). This was the equivalent to a change in 2.6 logits. Calculation for fractions of logits undertaken by a statistician indicated that in order to detect a change of 2.6 logits a minimum sample size of 17 per group was required. As the RCT design involves a two-group design, a total sample size of 34 was required to provide 80% power to detect clinically meaningful change between groups. Therefore, the study aimed to recruit 40 children to allow for a 10-12% drop-out rate.

### Outcome measures

Outcomes measures were completed on four occasions: baseline (1 to 2 weeks prior to injection), and at 1, 3 and 6 months after injection. Outcomes included measures from across the International Classification of Functioning, Disability and Health (ICF) [18] spectrum and involved a combination of investigator observed and parent report measures (See Table 1). This enabled analysis of the impact of intervention across the body function and activity domains.

**Table 1:** Outcomes classified according to International Classification of Functioning, Disability and Health (ICF)

Body Function	Activity	
	
	Capacity	Performance
modified Tardieu Scale (MTS), modified Ashworth Scale (MAS), Quality of Upper Extremity Skills Test (QUEST; dissociated movement subscale)	Quality of Upper Extremity Skills Test (grasps subscale)	Assisting Hand Assessment (AHA), Canadian Occupational Performance Measure (COPM), Goal Attainment Scaling (GAS), Pediatric Evaluation of Disability Inventory (PEDI), Pediatric Motor Activity Log (PMAL)

The effects of upper limb BoNT-A injection in both groups were monitored using measures of muscle spasticity and muscle tone including the Modified Ashworth Scale (MAS) and Modified Tardieu Scale (MTS).

### Primary Outcome

The primary outcome measure was the Assisting Hand Assessment (AHA) [51]. The administration of the AHA (Small Kids English version 4.4) was videotaped. The AHA is a standardized, criterion-referenced test for use with children aged 18 months to 12 years, who have a unilateral upper limb impairment [51]. It aims to measure how effectively a child uses their affected hand in bimanual play activities using 22 items. Unlike most other upper limb assessments for children with cerebral palsy, the AHA attempts to capture a child's typical performance when performing tasks rather than their best effort or "capacity" [51]. Change on the AHA is therefore more likely to reflect change in the child's upper limb use across multiple environments.

The AHA is conducted by video observation of the child involved in a 10-15 minute play session using the AHA test kit with specific toys. The twenty-two items defining different actions are then scored on a 4-point scale rating the quality of the performance. Four being effective, 3 somewhat effective, 2 ineffective and 1 indicating the child does not perform the action. The sum of raw scores (sum score) varies between 22 (low ability) to 88 points (high ability). Raw scores are converted to scaled scores ranging from 0 to 100. Using a computer generated logarithmic transformation, ordinal data are then converted to equal intervals in the unit logits [51]. The range of the AHA scale is -10.18 to +8.70 logits. Logit scores will be used in the analysis of data for this RCT.

The psychometric properties of the AHA have been described in several studies [51-54]. Using Rasch measurement analysis, validity and aspects of reliability were evaluated, with excellent results. There is strong evidence that AHA items measure a uni-dimensional construct with 95% of items fitting Rasch assumptions. Person response validity demonstrated that 97% of person's responses fitted the model. A person separation index of 6.16 demonstrated a very good ability to distinguish children of different ability levels [51,52]. A standard error mean of 0.28 (range 0.26-0.32) revealed the precision and reliability of the item measures [51]. More recently, reliability of the Small Kids AHA was found to be excellent with Intraclass Coefficients (ICC's) of 0.97 (20 raters) to 0.98 (2 raters) for interrater and for the intrarater 0.99 [53]. The same version also demonstrated excellent test-retest reliability (ICC 0.99). The Smallest Detectable Difference (SDD) over time indicated that a change in AHA scores from one test session to the next must be 3.89 sum scores (0.97 logits) or more to be considered a true change with 95% probability [54]. The AHA was initially designed for evaluation within a trial of CIMT [8] where it demonstrated responsiveness to change. Since then, studies evaluating HABIT [31,48] have also shown some change in the treatment group compared to the control group, providing evidence that the AHA was also responsive to change following bimanual intervention.

### Monitoring responses to Botulinum toxin-A injection

The Modified Ashworth Scale (MAS) is a 6-point, criterion referenced ordinal scale deigned to measure the resistance that is encountered when a limb is moved passively [55]. Scores range from 0 (no increase in muscle tone) to 4 (rigid). Movements assessed included: shoulder abduction and flexion; elbow extension and flexion; forearm supination and pronation; wrist extension and flexion; ulnar and radial deviation and thumb abduction.

The MAS has often been described in the literature as a measure of spasticity [55,56], however since muscle resistance in children with cerebral palsy is due to a combination of factors, only one of which is spasticity, the MAS is considered to have poor validity for spasticity evaluation [57]. Further, as there is no reference to the velocity of limb movement during examination, a central criterion in defining spasticity [3], the MAS is considered a measure of muscle tone. Following a review of papers across a broad range of diagnostic groups, Morris [58] estimated intra-rater reliability to be between 0.55 to 0.83 and 0.45 to 0.84 for inter-rater reliability. These estimations have been supported in recent studies in children with cerebral palsy where inter-rater reliability has been described as low [59,60]. To improve the reliability of using the MAS in this RCT, two physiotherapists, independent to the intervention component of the trial and experienced in administration and scoring of the MAS performed all assessments at a slow velocity. The MAS has demonstrated change in children following upper limb injection of BoNT-A combined with occupational therapy [24,25,61].

The modified Tardieu scale (MTS) was adapted from the original scale developed by Tardieu and colleagues [62]. The MTS grades the quality of the reaction of the muscle to passive stretch and measures the dynamic component of muscle spasticity [63]. This measure of spasticity is obtained when a joint is moved as fast as possible through its range of movement (V3 velocity) and the angle of "catch" elicited is measured using a goniometer. This is called R1. The difference between the angle of "catch" (R1) and the full passive range of movement (R2) reflects the potential range available in the joint if spasticity is eliminated [9,63]. The quality of the muscle reaction (resistance) is also rated when obtaining the R2 measurement, from 0 (no resistance through the course of the passive movement) to 5 (joint immovable).

In this RCT, administration of the MTS was jointly performed by an occupational therapist and physiotherapist both blinded to group assignment. Movements measured included: shoulder abduction and flexion; elbow extension and flexion; forearm supination and pronation; wrist extension and flexion; ulnar and radial deviation and thumb abduction. The velocity used to determine the angle of catch was V3 (as fast as possible), as recommended by Morris [58]. The MTS scale has demonstrated sensitivity to change when measuring spasticity following BoNT-A injection in the upper limb [24] and lower limb [63] in children with cerebral palsy. Despite this reported sensitivity, the MTS has demonstrated poor reliability as a measure of elbow flexor spasticity in children with cerebral palsy with large inter-sessional variation and difficulty in applying standardised velocities [64]. The MTS however, is still considered the only clinically valid measure of spasticity currently available [57,65]. To improve reliability of the MTS in this RCT, the same blinded raters (one occupational therapist, two physiotherapists) were used for each assessment occasion for each child.

### Secondary Outcomes

The secondary outcomes included measures of: quality of upper limb movement (Quality of Upper Extremity Skills Test (QUEST)); caregiver questionnaires relating to functional status of the child (Pediatric Evaluation of Disability Inventory (PEDI)) and perceived use of the affected upper limb (Pediatric Motor Activity Log (PMAL)); individual occupational performance (Canadian Occupational Performance Measure (COPM)); individual goal attainment (Goal Attainment Scaling (GAS)); muscle tone (the modified Ashworth Scale (MAS)); and spasticity (the modified Tardieu Scale (MTS)).

The Quality of Upper Extremity Skills Test (QUEST) is a descriptive, impairment-based measure designed to evaluate movement patterns and hand function in children with cerebral palsy [66]. The QUEST involves evaluation of 36 items of upper extremity function in four domains: dissociated movement, grasp, protective extension, and weight-bearing. For this RCT, both the affected and unaffected upper limbs were tested. Each item is scored as either a pass, fail or not-tested. A standardized score is obtained for each domain using the formula detailed in the manual [66] ranging from 0 (low ability) to 100 (high ability). A total score can be obtained by summing scores for each domain tested and dividing by the total number of domains tested. In this RCT, despite data for all domains being collected, only data from domains targeted by the interventions and therefore likely to change following mCIMT or BOT were analysed. These include grasp and dissociated movement. This is consistent with a more recent study evaluating the effects of BoNT-A and occupational therapy [23].

The QUEST is a reliable and valid measure for evaluating the quality of movement in children with cerebral palsy. Inter-observer ICC's range from 0.90 to 0.96 with test-retest correlations of 0.95 [67]. Currently, there is no evidence of the magnitude of change on individual QUEST domains required to determine clinical significance. The QUEST was originally designed to evaluate Neuro-developmental Therapy (NDT) [68]. It has since been widely adopted for use in trials evaluating BoNT-A and occupational therapy [23,24,61,69] and CIMT [35].

The Pediatric Evaluation of Disability Inventory (PEDI) [70] is a standardized assessment of how a child functions with an impairment in the context of their daily life. It has been standardized for children without impairment aged 6 months to 7.5 years and has established reliability and validity to detect the presence, extent and area of a functional delay in children with physical impairment or combined physical and cognitive impairment [71]. The PEDI is designed to measure a child's ability across 3 measurement scales: functional skills, caregiver assistance, and modifications used. Each scale is divided into 3 domains of self-care, mobility and social function, each of which can be administered separately.

Due to the nature of interventions provided in this trial being targeted at upper limb function rather than mobility and social function, only the self-care domain of the PEDI was administered. This is consistent with the use of the PEDI in many other trials evaluating BoNT-A and occupational therapy [23,25,26,72,73]. The 73 items in the functional skills scale are rated on a 2-point scale with 0 indicating inability to perform a tasks and 1 indicating a child is capable to perform the task. Eight items in the caregiver assistance scale are rated on a 6-point scale indicating the amount of assistance required to complete a task (0 = total assistance, 1 = maximal assistance, 2 = moderate assistance, 3 = minimal assistance, 4 = supervision and 5 = independent). Total raw scores are calculated by summing items from both the functional skills and caregiver assistance scales. Higher scores for functional skills level and caregiver assistance indicate better performance and increased independence. Normative standard scores and scaled scores are generated from the raw scores. Scaled scores range from 0 to 100, with increasing numbers representing increasing degrees of functional performance. Scaled scores were used in the data analysis for this trial.

PEDI scale construction was developed using Rasch measurement model and analytic techniques to evaluate construct validity and develop scaled scores. The PEDI has established validity [71,74-76]. High intra-rater reliability has been reported for self-care functional skills (ICC's 0.97 - 0.99) and self-care caregiver assistance (ICC's 0.94 - 0.99) [77,78]. Inter-rater reliability has been reported for self-care functional skills when administered as a parent interview [74,77]. In 2003, Iyer and colleagues reported that change scores of about 11% on the total scale appeared to represent clinically meaningful change [79]. Although the PEDI has been reported to show responsiveness to change over 6 months [80] more recently reported trials evaluating upper limb injection of BoNT-A in children with cerebral palsy, with or without occupational therapy, did not demonstrate a significant difference between groups [23,24,26,69,72]. As recommended by Berg et al. [78] to improve reliability, a single blinded assessor administered the PEDI in this RCT with the same parent across all time periods.

The Canadian Occupational Performance Measure (COPM) [81] is a client-centered measure designed to detect change in a persons' perception of their occupational performance in self-care abilities, productivity (i.e. for children school, pre-school activities) and leisure activities. It has adequate validity, adequate test-retest reliability (ICC of 0.63 for performance and 0.84 for satisfaction) and responsiveness to change [81-85]. The COPM has been used widely in intervention studies [85-88] and previously demonstrated responsiveness to change in studies evaluating BoNT-A and occupational therapy in children with cerebral palsy [23,24,61,72].

Due to the age of the children in this RCT, parental responses to the COPM were obtained rather than the child's. This adaptation has been supported in findings by Cusick et al. [89] who demonstrated acceptable internal consistency reliability for performance (mean alpha = 0.73) and satisfaction (mean alpha = 0.82), content and construct validity and responsiveness using this approach. The five most important occupational performance problem areas were selected using a 10-point scale, where 1 equals "not important at all" and 10 equals "extremely important". Performance in these areas was then rated by a parent on two scales: perception of their child's current performance and satisfaction with their child's performance. Ratings were again on a 10-point scale where scores closer to 10 indicated perceived better performances and increased satisfaction. At baseline, a total performance score was generated by summing the performance scores and dividing by the number of identified problems. Similarly, a total satisfaction score was generated by summing the satisfaction scores and dividing by the number of problems. These scores range from 1 to 10 [81]. Upon re-assessment, performance and satisfaction for each identified problem were again evaluated, scored from 1 to 10. Change in performance and change in satisfaction were calculated by subtracting Time 1 values from Time 2, 3 or 4 values. There is evidence that a change in summary scores (i.e. between initial and subsequent scores) of two or more is clinically significant [85].

The Goal Attainment Scale (GAS) is an individualized, criterion-referenced measure of treatment-induced change [90]. The GAS aims to measure an individual's success in achieving functional goals that have been determined prior to a treatment intervention. For this RCT, the three most important areas of occupational performance identified by parents using the COPM were used as the three nominated goals for scaling the GAS. During the baseline assessment session each goal was rated on a 5-point scale from -2 (current level of performance), -1 (less than expected outcome), 0 (expected outcome), +1 (more than expected outcome) to +2 (much greater than expected outcome). In the assessment periods during and after treatment (i.e. 1, 3 & 6 months post BoNT-A) parents were again asked by the blinded assessor to rate their child's performance in the three identified goals using the 5-point scale. Goals were not weighted and were therefore assessed as being of equal importance.

Using mathematical formulae, Kiresuk et al. [90] provide a method for summing the goals and converting them to a T-score. A T-score of 50 indicates that the goals were, on average, achieved [90]. Despite violating mathematical principles, most intervention trials using the GAS have adopted this methodology for analysis of GAS data. The T-score calculation implies data obtained from GAS are interval level and uni-dimensional in nature. This however, may not be the case and using these calculations when the assumptions are violated has been found to compromise the interpretation of change scores and confound the interpretation of parametric statistical tests [91]. In this RCT, the proportion of achieved goals was analysed. Achieved goals, that is those that change from -2 (baseline level of performance) to 0 (expected level of performance) will represent a clinically significant change.

The GAS has been used in five out of ten RCT's evaluating the use of BoNT-A and occupational therapy in children with cerebral palsy making it the most commonly used outcome measure across these trials. Despite its popularity and reported sensitivity to detect change, the validity and reliability of GAS is largely unknown [92]. Validity has been questioned, due to dependence on the skill of the therapists who scale the goals, their objectivity, and ability to select realistic goals and anticipate outcomes following a specific intervention [93]. With regard to sensitivity to change, Steenbeek et al. [92] reports that the responsiveness of GAS "depends on whether therapists and parents select goals and levels of attainment for each goal that represent clinically important changes in future performance" [[92], p. 553]. In this RCT, a single blinded assessor developed goals for each child and each goal was re-rated at each time point with the same parent.

The Pediatric Motor Activity Log [94] Version 1, is a parental rating on the frequency of use and quality of movement of the affected upper limb in 22 tasks.

Despite its use in studies evaluating CIMT [35,94], and a decision in 2003 to include the original version of the PMAL in this RCT, the measure has since been found to have inadequate construct validity and reliability [95]. Recent Rasch measurement modeling undertaken by Wallen and colleagues [95] found that the original scales of the PMAL had disordered rating structure. A revised version of the scale has been recommended which demonstrates strong test-retest reliability and adequate sensitivity to change. The new, collapsed scale however, may not adequately detect change in children at the extremes of ability and requires further exploration before it can be used as an outcome measure [95]. Unfortunately discussions with Wallen [95] indicated that transformation of the data from the original PMAL version obtained in this RCT into the revised PMAL would not be valid. This is because significant rewording of items and scoring criteria between versions has been required to validate the new tool. Based on Wallen and colleagues [95] findings of inadequate psychometrics of the version used in this RCT, PMAL outcomes will not be analysed or reported.

### Intervention

#### Botulinum toxin-A

Each child in the study received injections of BoNT-A (Allergan Australia P/L, Gordon, NSW, Australia) by a single, highly experienced rehabilitation specialist (HBR). Muscles injected were determined by the rehabilitation specialist in consultation with the chief investigator (BJH) at the Physical Rehabilitation clinic based on whether they appear to contribute to abnormal limb position and impair functional use of the limb. Injected muscles included *biceps brachii, brachialis, brachioradialis, pronator teres, pronator quadratus, flexor carpi ulnaris, flexor carpi radialis, flexor digitorum profundus, flexor digitorum superficialis, flexor pollicis longus, adductor pollicis, and opponens pollicis*. If indicated, children also received injections of BoNT-A into lower limb muscles during the same injection session. This was considered ethically appropriate for the child's overall management and was considered unlikely to interfere with upper limb outcomes. Injections were performed in theatre under a light general anaesthetic and all children were discharged on the same day. Muscle localisation was undertaken by the use of Teflon coated Botox injection needles (37 mm, 27 gauge) allowing electrical stimulation. Doses of BoNT-A were administered at a maximum dose of 15 U/kg (or 400 U). Dilution was 100 U/1 ml. During the first month following injection, the chief investigator (BJH) reviewed all children on two occasions to monitor the effect of the BoNT-A, identify potential adverse events and provide splinting intervention as required.

#### Splinting

Children with increased resistance to passive stretch or who exhibit early signs of muscle shortening were provided with a thermoplastic stretching splint designed for use overnight for a minimum of 6 hours per night. This protocol has been based on scientific support from a small number of animal studies reporting that muscles increase in length when immobilised in a lengthened position [96,97] and a few studies in adult lower limb literature that suggest a prolonged low load stretch is more effective than brief stretches in preventing contracture [98,99]. For children with cerebral palsy, evidence that static splinting maintains the mechanical-elastic properties of muscle remains weak [100]. Because passively positioning a joint during active movement and covering the skin is considered to limit the potential to strengthen antagonist muscles and impede sensory feedback from the hand, no day splints (i.e. neoprene, Second Skin(r), thermoplastic wrist cock-up) were used during enrolment in the RCT.

#### Movement-based therapy: General Considerations

One-month following injection of BoNT-A, children randomised to the experimental group received modified constraint-induced movement therapy (mCIMT). Children randomised to the control group received conventional bimanual occupational therapy (BOT). The individual-based treatment sessions of 45 to 60 minutes were conducted by the same occupational therapist (BJH) twice weekly for 8 weeks in an outpatient paediatric treatment room. In addition, children in the mCIMT experimental group were required to complete 3 hours of home program (with the mitt on), 7 days a week for the 8 week treatment period. Children in the BOT control group were also encouraged to undertake a home program but no time requirements were specified. A checklist was completed after each treatment session, identifying the activities used and general observations. Important general considerations for both groups related to establishing and maintaining rapport, equipment, preparing and implementing the sessions are described below.

Building rapport and establishing the therapist-child/therapist-parent relationship was a primary focus for initial treatment sessions. Along with identifying individual movement, hand skill and motor planning abilities of each child, this time allowed for selection of toys to match the demands of the task with the child's developmental level and specific hand skill goals. It was also necessary to establish expectations for future sessions, create an understanding of acceptable behaviour and to establish patterns of on-task behaviour for the child.

**Figure 1:**
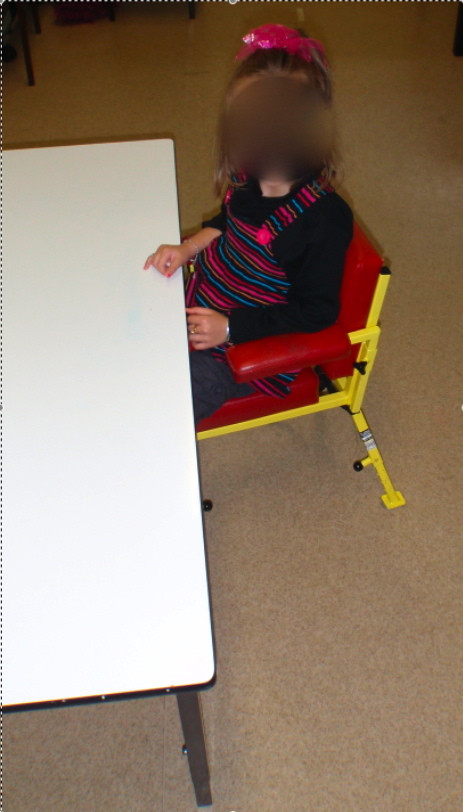
Table and chair used in therapy sessions

Most treatment was undertaken with the child sitting at a height adjustable table (See Figure 1). The therapist sat on a wheeled chair usually positioned on the child's affected side or behind the child. The child's chair, with footrest, armrest and pommel was adjusted so that the table was at waist height. This prevented the child from leaving the table to wander around the room or slip under the table. The therapist and child's position also: allowed the treatment to focus on hand skill development; provided adequate freedom of movement of upper limbs for reach and grasp; allowed visual monitoring of the hands during tasks; assisted in maintaining attention on the task; provided control of the child's immediate environment and; appropriately positioned the therapist to provide modelling of tasks and verbal and physical assistance with an emphasis on the required hand placement and movements, the task sequence and general demands of the task. Parents were seated opposite the child or on the child's unaffected side and beyond the child's reach.

Prior to each session a carefully selected range of fun and motivating toys were placed in the treatment room on an uncovered bench behind or to the side of the child's chair. To improve motivation the child was actively encouraged to choose the toys with which they wished to play. Periods without the child actively engaged in play were avoided to maintain motivation, attention and concentration. If the child became distracted, techniques such as using noise and sensory input (e.g. tapping the table with a toy, tapping the child's affected hand, raising volume of voice) were used to redirect their attention to the task. Because task repetition and practice are key components of training, any attempt by the child to prematurely stop task performance (i.e. by focussing on something else in the room) was ignored by the therapist and parent and the child's attention redirected to the task. The child was required to indicate when an activity had been finished through verbalisation or physical signs. If this occurred after only a few repetitions, further repetitions were negotiated before the activity is stopped. This was seen as important for compliance and motivation as the child felt included in the decision making process whilst further task repetition was achieved. No new activities were chosen until all parts of the activity had been packed up. This was crucial as repetitions of movement/hand skills achieved through packing up were often greater than in the activity itself.

With novel and/or challenging tasks, an inability to independently and successfully complete the demands of the task often leads to a child becoming disinterested or frustrated after only a few trials or repetitions. To facilitate learning, improve skills and task performance, develop resilience, and for the therapist to assist the child to establish patterns of on-task behaviour it was important not to allow the child to "give up" or avoid tasks. A variety of task-avoidant behaviours can be successfully used by children from a very early age. Initial and ongoing parent/therapist reinforcement can inadvertently condition task-avoidant behaviour, impeding the participation and engagement of the child and therefore the effectiveness of treatment. It was crucial for the therapist to establish a collaborative partnership with the parents to ensure consistent responses to these behaviours. On occasions where task avoidant behaviours became evident, the therapist provided advice and strategies to the parent(s) on appropriate responses to avoid reinforcement. Examples of typical task-avoidant behaviours displayed by children included:

• Throwing objects - this often occurs when a child does not understand the requirement of the task (poor motor planning) or the task is too challenging for the child's abilities. In this RCT the therapist always modelled novel task performance to demonstrate task demands. During early attempts at a task, significant physical assistance and/or verbal cuing were provided to ensure successful performance. The amount of assistance was then gradually decreased with future attempts. Any object thrown by the child was not collected by the therapist/parent and the behaviour was ignored. The object remained on the floor to ensure the child did not receive a response that reinforced the behaviour (i.e. stopping of activity, collection by parent). If the behaviour persisted the therapist pre-empted the throw preventing it from happening. The child's attention was redirected to the task and, if required, additional modelling of task performance or increased physical assistance was provided.

• Mouthing objects - young children can learn that mouthing objects leads to a quick and alarmed reaction from the parent/therapist that leads to diversion of attention away from the task. During treatment if a child demonstrated this behaviour, objects small enough for ingestion were avoided in all sessions until the behaviour ceased. To avoid encouraging this behaviour, larger objects were used (to ensure ingestion was not possible) and the behaviour was ignored when displayed. Any direct eye contact with the child from the parent and therapist was also avoided and directed to the task, so the child did not receive a response (i.e. facial expression from parent/therapist, verbal feedback).

• Crying can be an extremely effective strategy for task-avoidance by young children. The reinforcement of this behaviour occurs when the therapist immediately stops the task or parents intervene to provide comfort and reassurance through physical contact or removal of the child from the activity. The child can associate crying with successful task avoidance with resultant disruption to the session. During treatment, it was important for the therapist to quickly evaluate the situation. If the child was obviously distressed, parental comfort and reassurance was used to settle the child. Removal from the seat was avoided if at all possible. If the child was not obviously distressed and crying was deemed by the therapist/parent as task-avoidant behaviour the task was continued. A graded level of response was initiated: 1) child was not removed from position; 2) therapist negotiated with the child for one or two more repetitions prior to task completion; 3) therapist verbalised when task was completed 4) child was required to pack up the activity before choosing next activity; 5) if the child remained uncooperative the therapist and parent engaged in conversation or played the task/game whilst ignoring the child's behaviour; 6) if the child remained uncooperative the therapist asked the child if they would like the parent to leave the room; 7) as a last resort, if the child remained uncooperative the parent left the room until the child settled. As soon as the child settled the parent was invited back into the room. Great care was taken with this final response as it had the potential to cause significant anxiety and stress to the child. Over time, with consistent responses to crying from the parent and therapist, it was expected that the child would stop using crying as a technique for task avoidance.

#### Constraint-induced movement therapy

**Figure 2:**
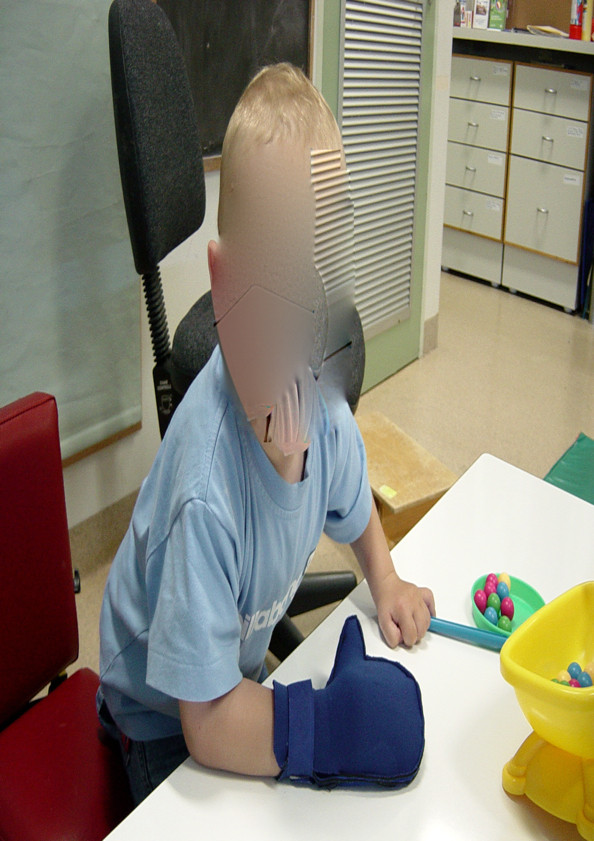
**Neoprene glove used in modified constraint-induced movement therapy**.

The modified constraint-induced movement therapy (mCIMT) protocol incorporated the two fundamental components of CIMT as described by Taub et al. [36]: the use of a restraint device (glove) and; the provision of massed practice to the affected upper limb (3 hours of home program). A comfortable neoprene (wet suit material) glove was worn on the hand of the non-affected upper limb to facilitate intensive practice of the affected upper limb. The neoprene glove, with a palmar thermoplastic insert over the fingers and thumb to prevent grasp, allowed the child to use the hand as an effective assist in bilateral activities, but did not allow active grasp of objects (see Figure 2). Importantly, the glove allowed the child to use the unaffected limb for breaking a fall, if needed. The intervention period was 2 months and involved wearing the glove 7 days per week. Children were expected to wear the glove for 3 hours per day, including therapy time and the home program, which could be split into different sessions of no less than 30 minutes duration. Families were expected to undertake an intensive home program of 3 hours per day. This could occur in the child's usual environment including home, crèche, preschool or school. Caregivers completed a log-book detailing the total period the restraint device was worn per day and any issues arising from use of the glove. The intention of the home program was to facilitate an intensive period of practice with the affected limb and to educate, empower and include families and caregivers in the treatment process. Families were provided with written and specific goals by the treating therapist after each session. These were based around development of specific hand skills such as grasp, hold, release, reach, in-hand manipulation. Families were encouraged to focus on these goals during the home program. Families were discouraged from placing the glove on the child without supervision to avoid frustration.

The implementation of mCIMT was based on the principles of motor-learning theory [30,101]. Eliasson [30] comprehensively describes the core principles of motor-learning including "learning to perform a task by developing strategies, learning a task by practicing skills and learning to use the hemiplegic hand through task practicing" (p. 56). Consideration of these principles highlights the important differences between adopting a motor learning approach within a CIMT protocol compared with a BOT protocol. Although both approaches use similar principles for development of motor skills (i.e. motivation and learning through repetitive practice), the uni-manual nature of CIMT only allows for practice of general aspects of hand function rather than the activity itself [30]. For the mCIMT group in this RCT, the uni-manual treatment focussed on repetitive practice of movements and skills with the affected limb (e.g. grasp, release, holding and transporting of balls into a ball tower, holding paint stamps and using adapted brushes, stacking blocks and other objects, holding a magnetic wands to catch a ball, magnetic fishing games, hammering games, Jenga). Using constraint of the unaffected upper limb and utilising games and play, uni-manual tasks were carefully selected to provide sufficient challenge and successful outcomes when using the affected upper limb. In doing so, learning was facilitated by practicing skills and the experience of using the hand through massed practice. It was not possible to target learning of bimanual strategies to achieve task performance.

Activities deemed too difficult by the therapist were avoided to prevent experiences of failure and frustration. This was particularly important in initial sessions where simple cause and effect activities were used (e.g. battery operated switch activated toys). Parents were educated that use of the glove must be perceived by the child as fun and enjoyable. Rewards, such as playing with favourite toys when the glove was used, was highly encouraged. Negative experiences, such as verbal threats and withdrawal of activities, were discouraged. The child was always kept busy and periods without the child actively engaging in play was avoided. If the child became distracted by the glove their attention was immediately redirected to the task by using noise and sensory input (e.g. tapping the table with a toy, tapping the child's affected hand, raising volume of voice). Any discussion regarding the glove was avoided. At the conclusion of each session the child was praised and informed the session had finished and the child was encouraged to independently remove the glove using his or her affected hand.

#### Bimanual Occupational Therapy

The bimanual occupational therapy (BOT) was underpinned by components of motor learning [102] and cognitive-based motor intervention [103,104]. This eclectic approach to treatment is commonly adopted by occupational therapists in the training of upper limb motor skills in children with cerebral palsy [30]. Details of the components adopted from motor skill acquisition, motor learning and motor control theory, the Assisting Hand Assessment hierarchy and cognitive based approaches are described below.

##### Motor skill acquisition, motor learning and motor control theory

Practical application of a *motor learning *framework requires implementation of a motor-teaching model whereby the therapist acts as a teacher and the child, a learner. Factors required to facilitate a child's learning of motor skills include: giving attention to the context; motivation and prior knowledge; instructions; modelling; taxonomy and sequencing of tasks; anticipation skills; mental and physical practice; repetition; facilitation-guidance; and feedback [102]. Similar core components have been outlined for improving motor skills in children using a *motor skills acquisition *frame of reference [27]. These frameworks [27,102], along with more recent advances in the knowledge of motor planning difficulties experienced by children with cerebral palsy [30,105], formed the core components of the BOT provided in this RCT. Examples of the practical implementation of these principles included:

• Initial and ongoing task analysis to identify if the child's performance was limited by execution of movement or motor planning difficulties (i.e. sequencing of movements) [30,105,106]. Motor planning impairment was observed by presenting a child with a novel task without prior modelling or task demonstration. It is evident when you know the child has the underlying physical capacity to complete the task but they simply cannot organise or plan the sequence of actions or required movements of the hands to successfully perform the task.

• Repetitive whole-task practice of challenging, motivating and purposeful bimanual activities (i.e. toys and games), carefully selected to facilitate learning and development of goal-based skills and independence with task completion [101].

• Use of modelling, physical assistance, verbal cues or environmental adaptation to enable the child to understand the critical features of the task and the environment.

• Facilitation of the child's learning and understanding of the role of their assisting hand (i.e. hemiplegic hand) using active problem solving.

• Grading of physical and/or verbal assistance provided to complete tasks.

• Provision of feedback focusing on the outcome, task and environment rather than specific movement performance.

• Provision of opportunities for the child to repetitively practice tasks in a range of contexts and environments.

##### Understanding and grading task difficulty in bimanual intervention

**Figure 3:**
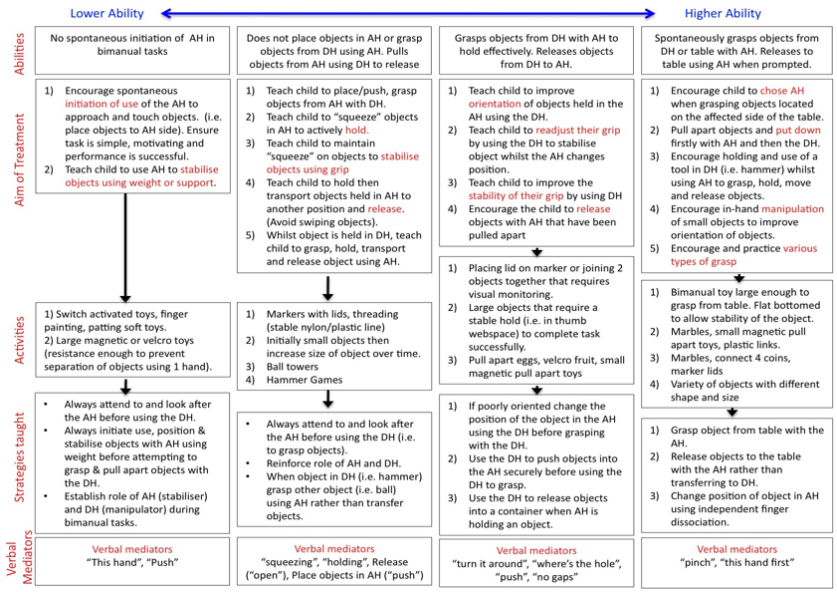
**Using the Assisting Hand Assessment hierarchy to grade treatment**. DH = Dominant Hand; AH = Assisting Hand

The Assisting Hand Assessment (AHA), designed specifically for children with unilateral impairment, was developed using a Rasch measurement model. This model allowed identification and ordering of the AHA test items on a scale from easiest to hardest. For example, simply approaching an item using the affected hand (easier item) to using in-hand manipulation skills to move objects in the affected hand (more difficult item). Based on the Rasch model, easier items are more likely to be easier to perform for all children than more difficult items. More able children are also more likely to perform better on more difficult items than less able children. The unique construct allows children's bimanual upper limb ability levels to be placed along a continuum of low ability to high ability. This knowledge can be used to design intervention and guide graded task performance [107]. For example, attempting to improve the bimanual performance of a child with low ability to place objects directly onto a table (most difficult item) using the assisting hand is inappropriate. Treatment for this child should focus on the development and consolidation of easier items such as holding objects or stabilising by grip. Conversely, continually focussing on easier items for a child with high abilities will not serve to improve their abilities on more difficult items. In this RCT, although specific details of the AHA scores remained unknown to the treating therapist, knowledge of the AHA hierarchy served as an important guide for selecting specific activities during the implementation of BOT. Treatment was tailored to the individual child based on their typical performance in the initial treatment sessions (see Figure 3).

##### Cognitive approach

Children with hemiplegic cerebral palsy have varied abilities to perform bimanual activities. Often children have the underlying motor abilities (i.e. adequate range of movement, ability to grasp, hold and release) to execute the required movements for task performance, however they are unable to independently identify the specific role for each hand, appropriately position the hands and object or sequence the movements or direction of force required to complete the task. These motor planning difficulties may be just as limiting for the performance of activities of daily living as is movement execution in children with cerebral palsy [105].

**Figure 4:**
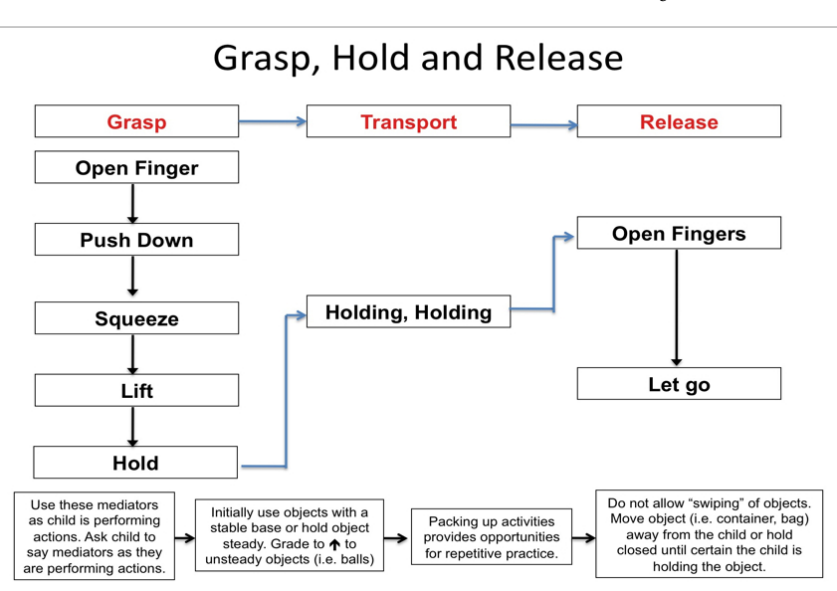
Development of grasp, hold and release using verbal mediators

**Figure 5:**
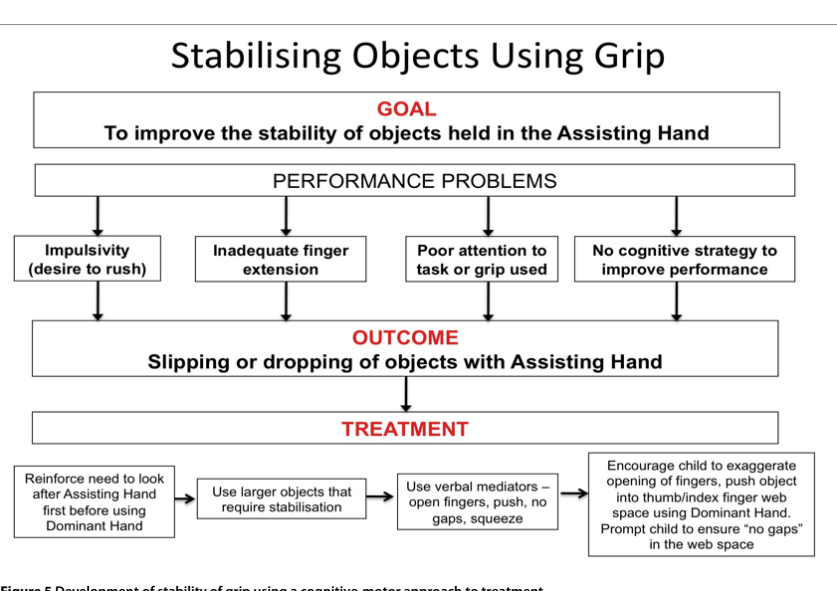
**Development of stability of grip using a cognitive-motor approach to treatment**.

Cognitive-based approaches [103,104] have evolved and been adapted for use in approaches such as Cognitive Orientation to daily Occupational Performance (CO-OP). Developed in the 1990's for the treatment of motor disorders in children with DCD [88,108], CO-OP is a task oriented problem-solving approach that utilises cognitive skills to improve occupational performance [109]. The child is guided to develop his/her own strategies based on problems encountered during a task. In this RCT, the bimanual approach to training was grounded in cognitive-based intervention theory. Much of the treatment targeted the motor planning abilities of children with both low and high bimanual abilities. Children engaged in a self-instructional training program that was carefully facilitated by the treating therapist [110]. In the context of this RCT, the treating therapist reinforced to children that the difficulties they often experienced with task performance was because they were not taking advantage of certain strategies or tricks that they could learn [110]. Novel activities were chosen which the child could only solve by carefully looking and listening, and for which a plan or strategy was required before any movement or action took place (e.g. pushing plastic links together). Attempts were made to encourage the child to plan ahead and reduce impulsive tendencies to quickly grab the object using their dominant hand before they thought about the role of affected hand in the task. Before handing the toy to the child, the therapist demonstrated exactly what was involved to complete the task. The therapist modelled the required movements and emphasised important sequences and strategies whilst verbalising these aloud using simple, key words. Deliberately, the therapist occasionally performed components of the task incorrectly and talked through how they could be corrected. The child was then handed the activity and encouraged to slowly perform the task using the sequences modelled by the therapist whilst using the same verbalisations, or verbalisations the child had developed. These verbalisations during task performance have been referred to as verbal mediation. The mediators serve to teach the child how to comprehend a task, direct motor movement through self commands and importantly, to guide, monitor and control their own performance [111]. If the child had difficulty performing a previously practiced task, the therapist prompted the child using recall of verbal mediators (see Figures 4 and 5). Importantly, this technique was demonstrated and reinforced to parents to ensure a similar process is undertaken in the home environment.

Identifying and breaking down the specific sequences of a task allowed the therapist to assist the child to discover deficient sequences and to prompt them to consider the error before the end of the whole task. This inhibits failure of the task at an incipient stage thereby reducing the likelihood that the child will become frustrated or non-compliant [111]. Typically, children become more motivated if they have been active and successful participants in the problem solving process. Over time, this approach aims to promote a child's resilience by independently prompting themselves to identify incorrect sequences before becoming angry or frustrated. With proficiency in performance the need for self-rehearsal diminishes [111]. Importantly, the child learns how to learn. In an attempt to facilitate generalisation of the learning, task practice in this RCT was encouraged in various environments (i.e. using home programs) and with different activities that required similar strategies [110].

### Statistical analysis

Data were managed and analysed using the Statistical Package for Social Sciences (SPSS version 16.0). Descriptive statistics were calculated to summarise the data set for both groups and to identify potential baseline differences between the groups; *p *values were used to indicate the strength of the evidence and will be interpreted according to Sterne and Davey Smith [112] Distributions of data from each group and for each occasion were assessed to determine if they met the assumptions for the various inferential analyses.

#### Testing the effectiveness of therapy (Hypotheses 1 and 2)

Using continuous, interval level data from the AHA, differences between the two groups were assessed using a linear regression approach to analysis of covariance (ANCOVA). This controlled for the covariates of baseline AHA scores and the child's age. These variables were included as covariates as they might provide alternative explanations for any observed changes in scores. The size of treatment effect was estimated by comparing differences in group means and their 95% confidence intervals. Regression analyses and scatter-plots were used to investigate the relationship of post-treatment outcome with initial deficit.

#### Testing the effect of therapy on secondary outcomes (Hypothesis 3)

Between group differences were assessed using ANOVA for continuous data (following tests of normality). Outcomes that had non-continuous data or did not meet usual assumptions of linear regression were investigated using non-parametric statistics.

#### Testing the clinical significance of the effect of therapy on primary outcome (Hypothesis 4)

The magnitude of treatment effect was evaluated relative to defined criterion of clinical significance.

#### Testing the effect of age and intensity of therapy (Hypotheses 5 & 6)

Data related to the number of minutes spent by all children undergoing treatment was extracted from the log book for each child. Associations between the intensity of therapy and outcome and age of children and outcome were determined, while controlling for the covariate of group, using ANCOVA.

### Safety evaluation

Each child was monitored throughout the trial period by the chief investigator. Following trial completion, all medical and research records were retrospectively audited. Any adverse events were recorded and classified according to whether they could be attributed to BoNT-A injection, general anaesthesia or movement-based treatment.

### Current study status

The study commenced recruitment in May 2003 and achieved target recruitment in September 2008. Participant follow-up was completed in March 2009. Data analysis is currently being undertaken.

### Amendments to the study since commencement (2003)

#### (1) Extension for completion date

Approval was granted by Southern Health and La Trobe University Ethics Committees to extend the completion date of the project to June 2010. Approval was requested due to the trial being behind schedule due to slow recruitment rates.

#### (2) Inclusion criteria

In March 2005 approval was granted by Southern Health and La Trobe University Ethics Committees to extend the upper age limit of children included in the trial from 4 to 6 years of age. Modification was requested due to the development of the upper age limit for the AHA, allowing the effective/valid measurement of children aged 4 to 6 years old. Children in this age range were also considered to potentially benefit from the treatments provided in the trial and therefore would be a clinical population these treatments would be offered to in the future.

## Discussion

This paper describes the methodology of a randomised controlled trial comparing the effects of modified constraint-induced movement therapy (a uni-manual therapy) versus conventional occupational therapy (a bimanual therapy) on improving bimanual upper limb performance of young children with hemiplegic cerebral palsy following upper limb injection of BoNT-A. The results of the paper will be disseminated through peer reviewed journal publications.

## Abbreviations

AHA: Assisting Hand Assessment; BoNT-A: Botulinum toxin-A; BOT: Bimanual occupational therapy; CIMT: Constraint-induced movement therapy; COPM: Occupational Performance Measure: GAS: Goal Attainment Scaling; HABIT: Hand arm bimanual intensive training; ICF: International Classification of Functioning, Disability and Health; MAS: Modified Ashworth Scale; mCIMT: Modified constraint-induced movement therapy; MTS: Modified Tardieu Scale; PEDI: Pediatric Evaluation of Disability Inventory; PMAL: Pediatric Motor Activity Log; QUEST: Quality of Upper Extremity Skills Test; RCT: Randomised controlled trial.

## Competing interests

Allergan Australia provided partial support by providing the BoNT-A (Botox(r)) used in the study, by payment of research assistants blind to group allocation, a blinded rater for the QUEST, and video editing services. The authors have no pecuniary interest in Allergan.

BJH is an occupational therapist and has received sponsorship from Allergan Australia to attend and teach at conferences and meetings but has no personal financial interest in Botox(r) or any related product.

CI is co-investigator of a randomised controlled trial investigating the effect of repeat injections of BoNT-A and occupational therapy in the upper limbs of children with hemiplegic cerebral palsy that has received support from Allergan Australia. In 2008, CI received a grant from Allergan Australia to present results of this trial at the American Academy of Cerebral Palsy and Developmental Medicine in Atlanta but has no personal financial interest in Botox^® ^or any related product.

HBR has received sponsorship from Allergan Australia to attend and teach at conferences and meetings but has no personal financial interest in Botox(r) or any related product.

LC - No competing interests

## Authors' contributions

BJH: PhD candidate, designed the RCT and wrote the paper. CI: PhD supervisor, designed the RCT and monitored progress revising the paper critically for intellectual content. HBR: monitored progress revising the paper critically for intellectual content. LC: PhD supervisor, designed the RCT and monitored progress revising the paper critically for intellectual content. All authors have read and approved the final manuscript.

## Pre-publication history

The pre-publication history for this paper can be accessed here:

http://www.biomedcentral.com/1471-2377/10/58/prepub
